# Comminuted Fracture of the Body of the Trapezium and Thumb Carpometacarpal Dislocation: A Particular Pattern

**DOI:** 10.1055/s-0038-1632406

**Published:** 2018-03-09

**Authors:** Laura Alonso, José Couceiro

**Affiliations:** 1 Hand Surgery Unit, Orthopedics Department. Hospital Marques de Valdecilla, Santander, Spain

**Keywords:** trapezium, carpometacarpal, joint dislocation, fracture

## Abstract

Trapezium fractures are uncommon injuries. Our case is that of a thumb carpometacarpal joint dislocation associated to a partly comminuted fracture of the body of the trapezium. To the best of the authors' knowledge, this particular variation has not been previously reported in the indexed literature.

Accounting for 3 to 5% of all carpal fractures, 1 2 trapezium fractures are an uncommon injury. These fractures are occasionally associated with other injuries such as Rolando fractures, Bennett fracture–dislocations, scaphoid fractures, and most infrequently to a dislocation of the thumb carpometacarpal joint. 1 3

In this article, we present a case of thumb carpometacarpal joint dislocation associated with a partly comminuted fracture of the body of the trapezium. To the best of the authors' knowledge, this particular variation has not been previously reported in the indexed literature, and it is complex to classify according to the criteria proposed by Walker et al. 4

## Case Report

Our patient is a 67-year-old woman, with a personal history of an Arnold–Chiari type I malformation. She was under treatment with risedronic acid and calcium by her family physician for a suspected diagnosis of osteoporosis.

She suffered a direct trauma to her left hand after a fall from standing height. ON her arrival to the emergency department, the patient complained of pain in her left thumb as well as functional impairment. Her thenar eminence was clearly swollen and deformed. Her neurovascular examination was unremarkable.

**Fig. 1:**
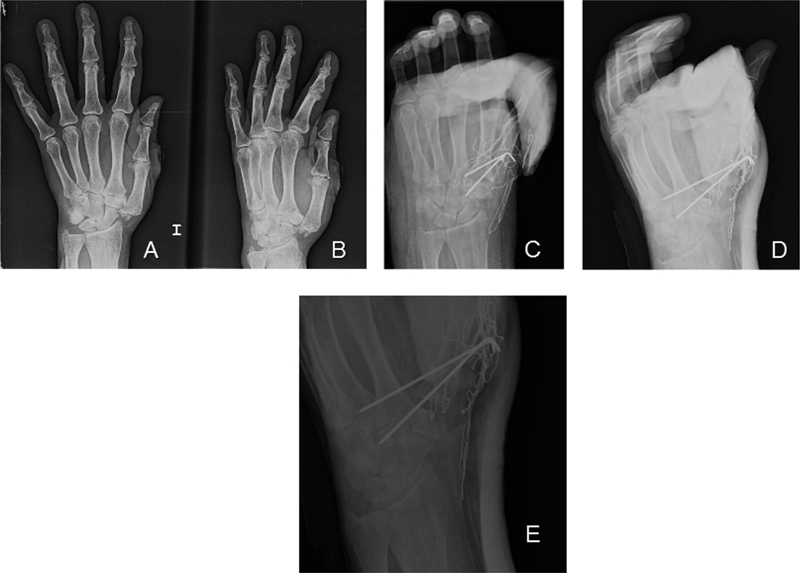
( **A** , **B** ) Simple X-rays of the fracture-dislocation. ( **C,D** ) Immediate postoperative X-rays following closed reduction and percutaneous stabilization with Kirshner wires. ( **E** ) The fracture-dislocation once reduced, the partly comminuted fracture pattern can be appreciated on this X-ray.

Simple X-rays of the affected thumb show a partly comminuted fracture of the body of the trapezium and an associated dislocation of the thumb carpometacarpal joint ( Fig. 1A , B ).

The patient was splinted and admitted for emergency surgery. The fracture–dislocation was then reduced in a closed manner and stabilized with two percutaneous Kirshner wires, one from the first to the second metacarpal, and the other from the first metacarpal to the trapezoid.

Postoperative X-rays can be seen in Fig. 1C and D .

A postoperative splint is maintained for the first 3 weeks. After this time, the splint was removed, and the patient was started on range-of-motion exercises.

The Kirshner wires were removed at 7 weeks.

At the latest follow-up, the range of motion of the interphalangeal and metacarpophalangeal joints of the thumb was measured with a finger goniometer (Exacta, North Coast medical, Gilroy, CA), the grip strength was measured with a Jamar dynamometer (Sammons Preston, Bolingbrook, IL), and pinch and key strength measurements were obtained with a hydraulic pinch dynamometer (Sammons Preston). Two-tip discrimination was obtained with a commercial two-tip discriminator (Arex Medical, Paris, France). Pain was scored on a visual analog scale (VAS) from 0 to 10. Thumb function was rated using the Kapandji scale from 0 to 10.

## Results

At 6 months follow-up, the patient referred no pain, and her VAS score was 0/10, both at rest and with exertion. Her two-tip discrimination was 6 mm. Her range of motion was 0/35 degrees at the metacarpophalangeal joint and 0/50 degrees at the interphalangeal joint of the thumb. Her grip strength was 20 kg, her key grip was 5.2 kg, and her pinch was 4.8 kg. The patient was able to touch the base of her small finger with her thumb tip, and her Kapandji score was 9.

**Fig. 2:**
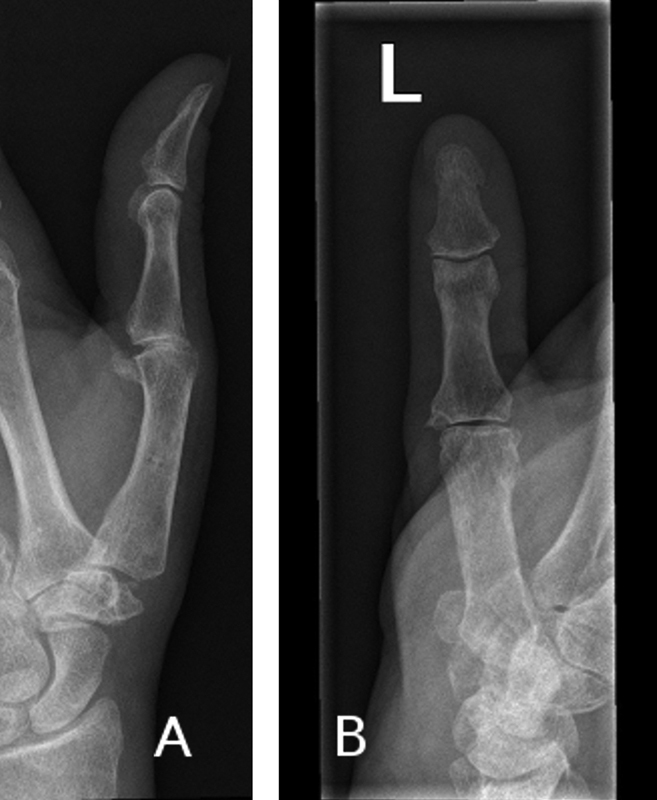
( **A** , **B** ) X-rays of the affected thumb at the latest follow-up.

She was completely satisfied with the result, she required no weight restrictions, and she had no difficulties in doing her regular housework or any other daily chores, which were mostly low-demand activities such as cooking and cleaning the house. She wanted no further procedures to be performed on her thumb. Final X-rays can be seen in Fig. 2A and B .

## Discussion

Although fractures of the body of the trapezium are uncommon injuries, their appearance in clinical practice is far from exceptional. A combination of these fractures with a dislocation of the thumb carpometacarpal joint is extremely rare, however. 1 2 3 Reports of such a combination are scarcely reported on the indexed literature. 1

Two main injury mechanisms have been proposed for thumb carpometacarpal dislocations. 1 In the first one, a dislocation occurs as a result of an axial overload on a flexed first metacarpal, and this pushes the metacarpal base dorsally on the trapezium, producing an injury to the carpometacarpal ligaments and finally a carpometacarpal dislocation.

The second injury mechanism involves an impact on the first webspace, and this produces a commissural shearing force. Depending on the force angle, a Bennett fracture–dislocation, a carpometacarpal dislocation, or a trapezium fracture occurs.

In case a fracture of the trapezium is generated, it can be classified into five groups, according to Walker et al: 4 type I is a horizontal fracture of the trapezium, types IIa and IIb involve the anterolateral trapezium ridge, type III involve the anteromedial trapezium, type IV are vertical trapezium fractures, and type V are comminuted fractures of the body of the trapezium.

According to this classification, our case should be classified as a type V. The fracture comminution affected only approximately 50% of the trapezium in this case, however ( Fig. 1E ). It is likely that this allowed a closed reduction of the fracture–dislocation and an acceptable result following stabilization with percutaneous Kirshner wires.

Our case exhibits therefore certain unique features. The fracture pattern of the trapezium is not a type I or IV since there is not a single fracture line, with big fragments, which would make internal fixation easier or at least possible. It is not an isolated fracture of the ridge and therefore would not fit in group II or III. We have not found any published reports of type V fractures associated with thumb carpometacarpal dislocations, and this may be due to the fact that if there is a comminuted fracture of the trapezium, the metacarpal has no longer a base to dislocate from. If there is a partial comminution, as in our case, the metacarpal may be still dislocated from the remaining trapezium.

We can only hypothesize that if an axial force is applied to the thumb metacarpal, and this metacarpal is in a certain abduction or adduction position, the subsequent trapezium fracture, if it is produced, may be a partly comminuted type V fracture. If the force is maintained, the metacarpal can be dislocated from the trapezium, creating a fracture dislocation pattern with the particularities that have been formerly described. This represents a variation of the fifth type of fractures described by Walker et al, which, in this case, was associated with a thumb carpometacarpal dislocation. To the best of the authors' knowledge, this combination has not been previously described on the indexed literature.

The partial conmminution allows for its closed reduction and percutaneous fixation, making difficult or even precluding, however; any attempts at rigid internal fixation with devices such as screws or plates.

Our case had a favorable outcome after surgery, and early recognition and treatment of the injury played an important role in achieving this result.

A late diagnosis of this fracture–dislocation, due to a delayed presentation of the patient to the emergency department or due to the diagnosis being missed by the treating physician, has a high chance of severely impacting thumb function owing to the important injury to the articular cartilage and the ligaments of the first carpometacarpal joint. If our group were to face this specific clinical scenario, we would probably opt for a thumb carpometacarpal fusion with a plate and screws, or a trapezium resection and tendon interposition procedure.
